# The Relationship between the Restorative Perception of the Environment and the Physiological and Psychological Effects of Different Types of Forests on University Students

**DOI:** 10.3390/ijerph182212224

**Published:** 2021-11-21

**Authors:** Qiaohui Liu, Xiaoping Wang, Jinglan Liu, Guolin Zhang, Congying An, Yuqi Liu, Xiaoli Fan, Yishen Hu, Heng Zhang

**Affiliations:** 1 College of Forestry, Beijing Forestry University, Beijing 100083, China; liuqiaohui202109@163.com (Q.L.); wangxp@bfdic.com (X.W.); 2 School of Ecology and Nature Conservation, Beijing Forestry University, Beijing 100083, China; acy19961219@163.com (C.A.); rachelyuqi@gmail.com (Y.L.); fanxiao0802@163.com (X.F.); hys16637102261@163.com (Y.H.); 3 Management and Protection Center, Xiaolongshan National Nature Conservation, Tianshui 741020, China; z13884583683@163.com; 4 Ming Dynasty Tombs Forest Farm, Beijing 102200, China; zhyxm2003@163.com

**Keywords:** restorative environment, forest types, forest therapy, physiological restoration, psychological impact, human well-being

## Abstract

Short-term exposure to a forest environment is beneficial to human physiological and psychological health. However, there is little known about the relationship between the restorative perception of environment and physiological and psychological restoration achieved by experiencing the forest environment. This study evaluated the relationship between the restorative perception of different types of forests and human physiological and psychological effects. A sample of 30 young adult students from Beijing Forestry University was exposed to coniferous, deciduous, and mixed forests as well as an urban site. Restorative perception of the environment was measured using the PRS questionnaire. Restorative effects were measured using physiological indicators (blood pressure and heart rate) and three psychological questionnaires (Restorative Outcome Scale; Subjective Vitality Scale; Warwick–Edinburgh Mental Well-being Scale). The results demonstrated the following: (1) There were significant differences in the perceived restorative power of the three types of forests, with the highest level in the mixed forest, followed by the coniferous forest and the deciduous forest. (2) All types of forests were beneficial to physiological and psychological restoration. The mixed forest had the greatest effect in lowering blood pressure and heart rate as well as increasing vitality, while the coniferous forest had the strongest increases in psychological restoration and positive mental health. (3) The level of perceived restorative power of environment was positively related to the physiological and psychological restoration. These findings provide practical evidence for forest therapy that can maximize the restorative potential of forests.

## 1. Introduction

Industrialization has greatly promoted the emergence and development of modern cities, and urbanization has further expanded the spatial scope of cities. As a result, the original natural environment and human settlement environment have undergone drastic changes. Fast-paced urban life, intense competitive pressure, busy work, and separation from the natural environment have become incentives for an increasing number of chronic diseases or mental illnesses such as diabetes, obesity, insomnia, and depression among urban residents [1,2,3]. Moreover, studies in growing numbers have confirmed that people can obtain restorative experiences in the natural environment and that there is a proven positive relationship between exposure to nature and health-related well-being [4,5]. The natural environment is considered as a restorative environment for most people, which can help people to achieve a better recovery from mental fatigue and negative emotions as well as physiological reactions [6,7].

Attention restoration theory (ART) and stress reduction theory (SRT) are usually seen as essential theories to illustrate the pathways toward restoration in nature. ART is based on the assumption that nature can prompt the individual to transform from “directed attention” to “undirected attention”, so that the individual can be replenished from the attention fatigue caused by “directed attention”, that is, the stress state can be restored during the interaction between people and nature [6]. SRT is based on the point of psychological evolution, which states that people have dependence on or preference for certain natural characteristics under stress, and contact with the natural environment helps people gain recovery from all stress [7]. Both ART and SRT theories posit that people have a strong and consistent positive trend toward the natural environment. According to ART, a restorative environment generally has four characteristics, namely, being away, fascination, extent, and compatibility [6]. Being away refers to the psychological state that is generated by a situation that is different from the usual situation, which can avoid the fatigue of the psychological state [8]. Fascination means the environment is so attractive that contextual information can be noticed without effort, so as to avoid continuous use of attention in order to get relaxion [9]. Extent indicates the environment has enough richness and continuity of scenes so that concentrated attention can be rested. Compatibility requires an environment having a good match with the individual’s motivation, behavior, information, ability, or inclinations [10]. Hartig designed and developed the Perceived Restoration Scale (PRS) that is used to measure the restorative potential of an environment based on the ART [8], which has been widely used in an increasing number of environmental restoration studies.

The effects of a restorative environment are reported in both physiological restoration and psychological well-being. Numerous studies have confirmed that restorative environments are beneficial for improving the cognitive and positive emotional state [11,12] and reducing anxiety and stress [13,14] as well as lowering the occurrence of physical diseases [15], promoting self-identity [16], stimulating creativity [17], restoring attention [18], and boosting people to achieve a better mental state and work performance. Meanwhile, studies have concluded that there were differences in the restorative effects of individuals in different types of environments [18,19]. It was found that scenes in daily life in a city with more natural elements achieved a higher perception of restoration [20]. In addition, the dimensions of restorative perception of the environment were of different levels of importance [21]. Some studies stated that the fascination dimension had the greatest influence on perceived restorative power of the environment [22,23], while another confirmed that compatibility had the greatest impact [24]. Moreover, researchers have established a positive relationship between the perceived restorative power of the natural environments and physiological and psychological responses, which means that a higher restorative perception indicates a stronger physiological and psychological restorative effect [25].

Forests are recognized as one of the core health resources because they provide excellent ecological environments and rich scenic views that create a safer and more relaxing environment than other types of vegetation [26]. At present, most scientific research mainly focusses on the difference in the effect of the urban–forest dichotomy, and results proved the good performance of forests in relation to health benefits [27]. Exposure to a forest environment helps in reducing blood pressure [28,29] and heart rate [30] while increasing feelings of vitality, subjective revival, and mental health [31,32]. However, studies have found that not all forests have the same level of restorative effects [33] as the promotional effects of the forest on a subjects’ health might be influenced by the different forest characteristics (age, tree species, size, type, and density of trees and vegetation) that shaped the forest landscapes and environments [27]. Even the shape of the trees in the forest can affect health benefits, trees with a natural shape had a better positive effect on lifting emotions and lowering blood pressure than trees with geometric shapes [34]. Furthermore, it was confirmed that the restorative effects were closely related to the abundance of plant species in the natural environment [35], whereas excessive naturalness and plant density were also not conducive to the restorative effect. A field experiment in Switzerland showed that walking in a dense forest with obvious signs of maintenance was more restorative than walking in an equally dense but unkempt forest [36]. Therefore, the capacity of forests to improve human health may be influenced by different forest characteristics, which means that some forests are more appropriate or effective than others.

According to arousal theory [37], people’s cognition and evaluation determine the emotional forms, and the intensity of emotions depends on the level of arousal. Evaluation and emotional forms work together on the state of emotions, and the initiation of mood is usually a response to environmental stimuli and emotions [38]. The cognitive theory of emotion posits that when people receive stimuli in the environment, they will also adjust and adapt [39]. Based on above-mentioned theory, Bagozzi put forward the self-regulation of attitudes theory, which states that evaluation will promote the generation of emotions and further affect the individual’s behavior or behavior intention, which is generally expressed as a process of evaluation–emotion–behavior [40]. The restorative perception of the environment is also a gradual process of psychological perception, which proceeds from cognitive evaluation of the environment to physiological and psychological reactions. It is easy for people to rely on and identify with a well-evaluated environment and realize the match between relaxation expectations and environment to promote the recovery of physical and mental health.

However, to our knowledge, even though a few studies have explored the restorative effects of forests on human health and well-being, few have examined the relationship between the restorative perception assessed using the Perceived Restoration Scale (PRS) and physiological and psychological restoration achieved by experiencing the forest environment. Therefore, the main objective of this study was to investigate the relationship of the restorative perception of different types of forests (mixed forest, deciduous forest, and coniferous forest) and its human physiological and psychological restorative effects. In particular, we sought to pinpoint the relation between restorative perception of environment and physiological and psychological effects by answering three research questions: (I) Would the restorative perception assessment of different types of forests be different? (II) Do different types of forests influence physiological and psychological restoration differently? (III) Would the relationships between restorative perception and physiological and psychological restoration be positive?

## 2. Materials and Methods

### 2.1. Participants

Participants were recruited from Beijing Forestry University by the Internet (WeChat mobile application). There were 30 adult university students who participated in the field experiments, including 16 female and 14 male participants with a mean age of 23.9 years (SD = 1.86). The sample included different students with majors to avoid the bias of the students’ major in regard to their forest science knowledge. All subjects of this study were healthy Chinese adults. Participants with physical or mental disorders or those taking insomnia drugs or stimulants as well as those with heavy smoking and drinking habits were excluded from the study. During the recruitment, the aims and experimental procedures of the study were informed to the students who willingly participated. All procedures involved in this study were in accordance with the ethical standards of the Ethics Committee of Beijing Forestry University and the Declaration of Helsinki from 1964. All participants gave written consent for their voluntary participation.

### 2.2. Study Sites

**Figure 1:**
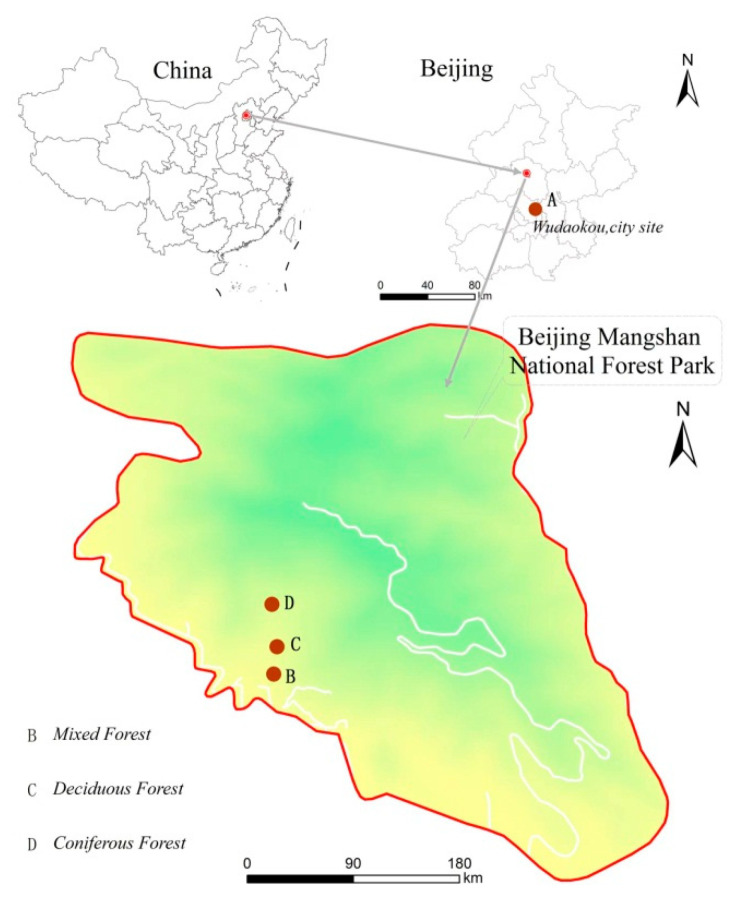
The map of experimental locations.

Participants were exposed to three different types of forests and an urban site located in Beijing, China (Figure 1). All the forest sites were in Mangshan National Forest Park, the largest national forest park in Beijing, which is 40 km away from the city. The total area of the Mangshan National Forest Park is 8622 ha, and the forest coverage rate is 86%. The urban site was a city square in the center of downtown.

**Figure 2:**
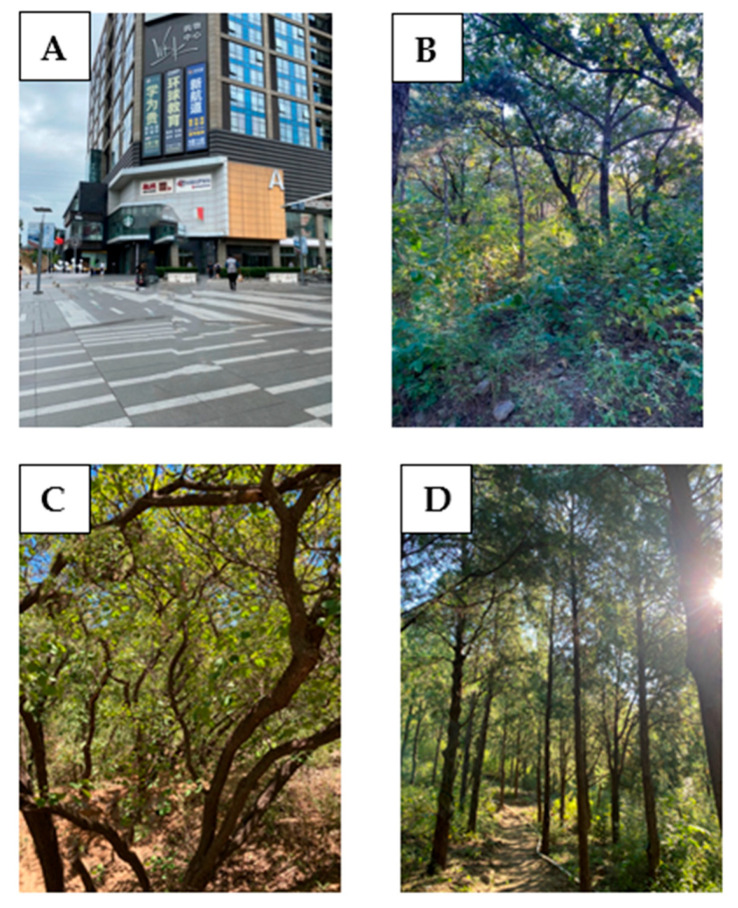
Photos: (**A**) city site, (**B**) mixed forest, (**C**) deciduous forest, (**D**) coniferous forest.

The urban site was Wudaokou Shopping Square, which is a city square in the center of downtown with a large amount of people and vehicles, and the surrounding area was characterized by urban elements such as a subway station, a shopping mall, roads, supermarkets, and community houses, which is a typical urban site (Figure 2A).

The forest site in which the species composition was dominated by *Quercus mongolica* and *Pinus tabuliformis* was coded as a mixed forest. It is a typical coniferous and broad-leaved mixed forest (Figure 2B). For the second forest site, the dominant tree species was *Cotinus coggygria*, which grows in summer and sheds its leaves in winter; it was coded as a deciduous forest (Figure 2C). The species composition of the third forest site was dominated by *Platycladus orientalis*, evergreen forest, which was coded as a coniferous forest (Figure 2D). All three types of forests were planted about 40 years ago, and forest characteristics in forest sites investigated by the study team are shown in Table 1.

### 2.3. Procedures

**Figure 3:**
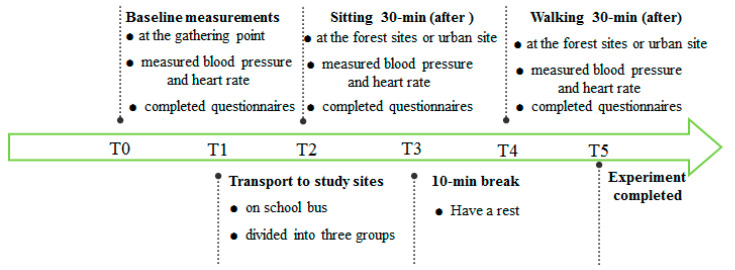
Flow diagram for on-site experimental protocol.

The experiment took place in June 2021 in sunny weather conditions. Participants were exposed to each of the study sites at the same time of a different day. The study protocol was the same for each experiment (Figure 3). Participants gathered in a classroom of Beijing Forestry University at 8 a.m. of each study day and had their blood pressure and heart rate measured and completed all questionnaires at the gathering point as baseline measurements. The procedure of obtaining baseline measurements took nearly 10 min. Afterward, participants were transported to the study sites by a school bus (nearly 40 min). While on the bus, participants were randomly and evenly divided into three groups, with 10 participants in each group (group A, group B, and group C). The participants were asked to sit at the selected spot for 30 min in the respective forest sites. After sitting, researchers conducted blood pressure and heart rate measurements, and then the participants finished the questionnaires at the sitting sites. After taking a break for 10 min, participants were asked to walk 30 min with a speed of moderate energy expenditure. After walking, the participants chose a flat and comfortable place to sit down and rest for 3 min, researchers conducted blood pressure and heart rate measurements, and then participants completed the questionnaires. The entire experimental procedure required each participant to visit four study sites including three forest sites (three different types of forests) and one urban site. Each person only visited one forest site on one day. All participants visited three forest sites and an urban site in order to increase the validity of the study. All 30 participants visited the urban site at the same time of day and were evenly and randomly divided into three groups guided by three researchers in each group in order to conduct the measurements. During the experiment period, talking, eating, and drinking energy drinks were not allowed.

### 2.4. Measurements

Blood pressure (systolic blood pressure (SBP), diastolic blood pressure (DBP)) and heart rate (HR) were measured to record participants’ physiological responses. Heart rate and blood pressure are closely related to the sympathetic and parasympathetic nervous system in the autonomous nervous system. When an individual is in a state of rest or relaxation, the function of the parasympathetic nervous system is strengthened, and the heart rate slows down while blood pressure decreases. Conversely, when an individual is in a state of tension or stress, the excitability of the sympathetic nervous system increases, and the heart rate and blood pressure also increase [41,42]. Blood pressure parameters were measured using an Omron IntelliSense blood pressure monitor (HEM-7207). Heart rate was measured using a Polar monitor (TEAM PRO). A lower score indicates higher physiological well-being.

The Perceived Restoration Scale (PRS) was used to measure the restorative perception of the environment, which includes four sub-scales, namely being away, fascination, extent, and compatibility [8]. In this study, we used the scale with 22 items modified by Chinese scholars [43]. Each item had a seven-point Likert scale ranging from 0 (= totally disagree) to 6 (= totally agree). A higher score indicates higher restorative potential.

Three psychological measurement questionnaires all in their Chinese versions were used to measure participants’ psychological responses. The Restorative Outcome Scale (ROS) was used to evaluate the restorative effects and was composed of six items. Each item was ranked on a seven-point Likert scale ranging from 0 (=totally disagree) to 6 (=totally agree). In this study, we used the scale modified for forest-related experience [32]. The Subjective Vitality Scale (SVS) was used to the measure level of vitality. It was composed of four items with a seven-point Likert scale ranging from 0 (=totally disagree) to 6 (=totally agree). The reliability and validity of the SVS have also been confirmed in a previous study [44]. The Warwick–Edinburgh Mental Well-being Scale (WEMWBS) was used to assess positive mental health. This scale can evaluate the positive emotions of mental health and satisfaction in interpersonal relationships [45]. The Chinese version has 14 items and its reliability and validity have been verified in a previous study [46]. Each item was evaluated with a five-point Likert scale ranging from 0 (=not at all) to 4 (=extremely). A higher score indicates higher psychological well-being in the three psychological measurement questionnaires. During the site study, 10 min was enough for each participant to finish all these questionnaires. All questionnaires in this study used the time frame “at this moment” for timely measurements of the participants’ responses.

Although PRS could also be categorized as a psychological assessment scale, there are distinct differences between PRS and other three psychological assessment scales. PRS is the measurement used to assess whether the environment is a restorative environment, which focus on evaluation of the environment itself. As other three psychological questionnaires (Restorative Outcome Scale; Subjective Vitality 24 Scale; Warwick–Edinburgh Mental Well-being Scale) were used to measure the reactions or responses of participants to the investigated environments, which are more likely to be used for timely measurement of participants’ psychological responses (or known as restorative effects) as influenced by the environment.

### 2.5. Statistical Analysis

In this study, we used Excel 2010 (Microsoft Corporation) to record raw data from all questionnaires and measurements. All statistical analyses were processed by SPSS 23.0 (IBM, Armonk, NY, USA). We used a one-way analysis of variance (ANOVA) to compare the differences between the values of the physiological, psychological, and perceived restoration level among the three types of forests and the urban site. Least significant difference (LSD) was used to do a post hoc test to compare the differences of variables in different types of forests. Spearman rank correlations analyses were conducted to explore the relationship between the restorative perception of environment and the physiological and psychological effects.

## 3. Results

### 3.1. Differences in the Restorative Perception of Experimental Sites

#### 3.1.1. The Total Scores of PRS in the Different Types of Forests and the Urban Site

A one-way ANOVA analysis revealed that the PRS scores significantly differed among the four experimental sites (F = 1268.04, *p* < 0.001). The mixed forest was perceived as the most restorative environment, followed by the coniferous forest and the deciduous forest, and the urban site was the least restorative (Table 2). The LSD post hoc test showed significant differences between the urban site and the three types of forests (*p* < 0.001), and there were also significant differences among the three forest sites (*p* < 0.05).

#### 3.1.2. The Four Dimensions of PRS among the Different Types of Forests and the Urban Site

**Figure 4:**
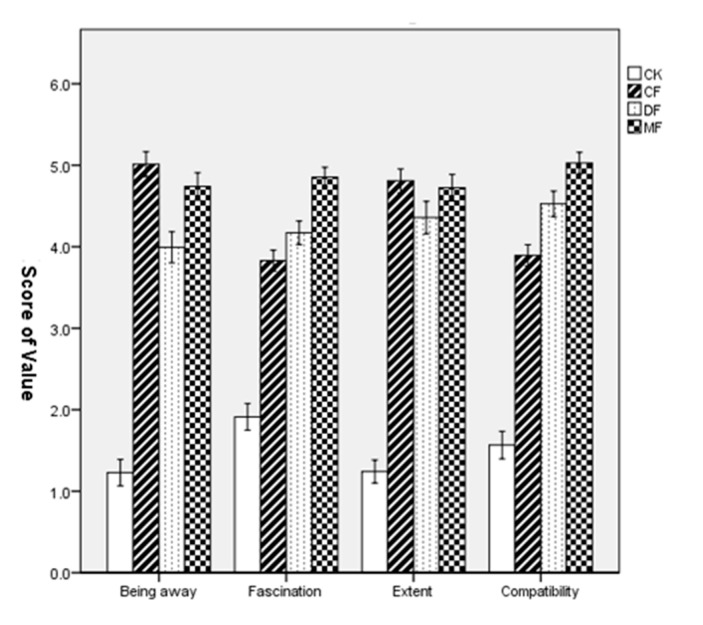
Comparison of the dimensional scores of PRS in four environmental sites. Data are presented as the means ± SEs. CK = city site, CF = coniferous forest, DF = deciduous forest, MF = mixed forest.

The scores in the four dimensions of PRS showed that all three forest sites had higher values than the city site. For the “fascination” and “compatibility” dimensions, the mixed forest was at the highest level, followed by the deciduous forest and the coniferous forest. For “being away” and “extent” dimensions, the coniferous forest was at the highest level, followed by the mixed forest and the deciduous forest (Table 2, Figure 4).

A one-way ANOVA revealed that all four-dimensional scores of “being away” (F = 410, *p* < 0.001), “fascination” (F = 312, *p* < 0.001), “extent” (F = 422, *p* < 0.001), and “compatibility” (F = 418, *p* < 0.001) were significant. The LSD post hoc test showed significant differences in the dimensions of “being away”, “fascination”, “extent”, and “compatibility” between the urban site and the three forests (*p* < 0.001), and there were also significant differences among the three forest sites (*p* < 0.05).

### 3.2. Differences in the Physiological Restoration in Three Types of Forests

**Figure 5:**
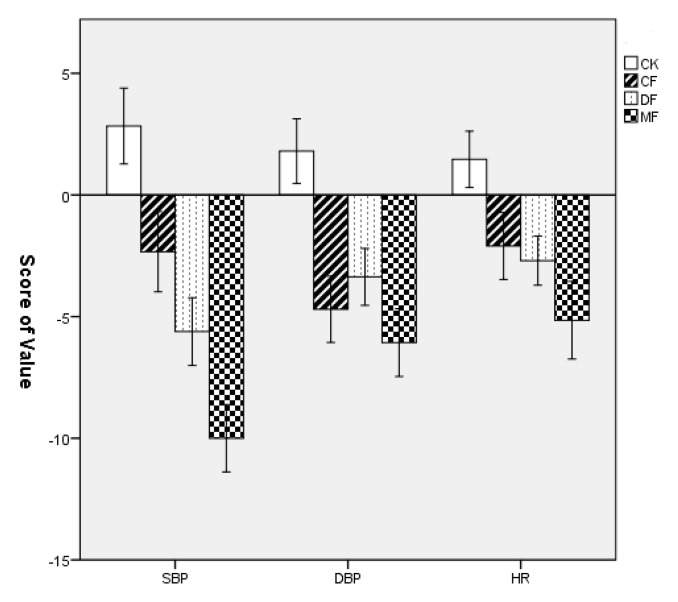
Comparison of reduction in values of SBP and DBP as well as HR in the four experimental sites. Data are presented as the means ± SEs. CK = urban site, CF = coniferous forest, DF = deciduous forest, MF = mixed forest.

Table 3 shows the mean values and standard deviation (SD) of the evaluated physiological parameters, including blood pressure and heart rate. The mean SBP, DBP, and HR values decreased in all forest sites, while these parameters had increases in the urban site, with the strongest decrease in the mixed forest and the lowest decrease in the deciduous forest (Table 3, Figure 5). The results of a one-way ANOVA showed that the restoration effects on SBP (F = 52.13, *p* < 0.001), DBP (F = 27.28, *p* < 0.001) and HR (F = 17.61, *p* < 0.001) differed significantly among the three forest sites and the urban site (Figure 4). The LSD post hoc test showed significant differences in SBP, DBP, and HR values between the urban site and the three forest sites (*p* < 0.001), and there were also significant differences among the three forest sites (*p* < 0.05).

### 3.3. Differences in the Psychological Restoration in Three Types of Forests

**Figure 6:**
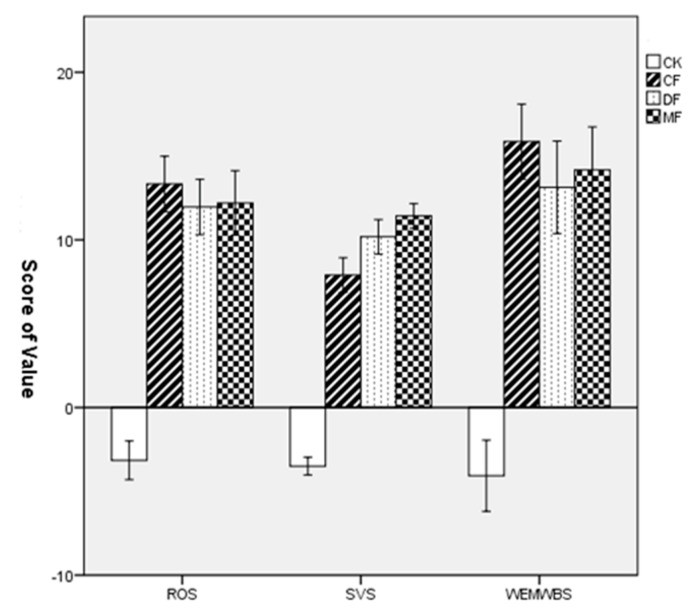
Comparison of the increased values of ROS, SVS, and WEMWBS in the four experimental sites. Data are presented as the mean ± SEs. CK = city site, CF = coniferous forest, DF = deciduous forest, MF = mixed forest.

Table 4 illustrates the mean values and standard deviation (SD) of the assessed psychological parameters, including ROS, SVS, and WEMWBS measures. The mean ROS, SVS, and WEMWBS values increased in all forest sites, while they decreased in the city site. The mean value of ROS and WEMWBS increased the most in the coniferous forest followed by the mixed forest and the deciduous forest. The highest scores of SVS were achieved in the mixed forest (Table 4). The results of a one-way ANOVA showed that restorative effects on ROS (F = 302.71, *p* < 0.001), SVS (F = 328.24, *p* < 0.001), and WEMWBS (F = 293.47, *p* < 0.001) differed significantly among the three forest sites and the urban site (Figure 6). The LSD post hoc test showed significant differences in ROS, SVS, and WEMWBS values between the urban site and the three forest sites (*p* < 0.001), and there were also significant differences among the three forest sites (*p* < 0.05).

### 3.4. The Relationship between Restorative Perception of Environment and Physiological Parameters

The Spearman rank correlations showed that the level of PRS and the four sub-scales of PRS were positively related to the restoration experience measured by the SBP, DBP, and HR in all forest environments (Table 5). The correlation coefficient is a negative value, but it is described as a positive correlation because decreases in physiological indicators portray a better restorative effect of the environment. The highest correlations were calculated for the PRS in the SBP, and the highest correlations were calculated for the four sub-scales of PRS in “compatibility” followed by “fascination” in the SBP. All these correlations between physiological indicators and PRS as well as four sub-scales of PRS were significant (*p* < 0.01).

### 3.5. The Relationship between Restorative Perception of the Environment and Psychological Parameters

The Spearman rank correlations showed that the level of PRS and the four sub-scales of PRS were positively related to the restoration experience measured by the ROS, SVS, and WEMWBS in all forest environments (Table 6). The highest correlations were calculated for the PRS in the SVS, and the highest correlations were calculated for the four sub-scales of PRS in “being away” in the ROS. All these correlations between physiological indicators and PRS as well as the four sub-scales of PRS were significant (*p* < 0.01).

## 4. Discussion

### 4.1. Restorative Perception of Different Environments

There were significant differences in the values of the PRS and the four sub-scales of the PRS in the different types of forests and the urban site. The mean scores of the PRS were at a higher level in all forest sites compared with that in the urban site, which means that the forest environments were better restorative environments. This is consistent with previous research on restorative forest environments [47,48,49]. The mean scores of PRS had the highest values in the mixed forest followed by the coniferous forest and deciduous forest. A possible explanation for the results may be the public’s preferences for the structural attributes of forests. To some degree, people seem to prefer natural landscapes. The scenes in the less natural group received less visual attention from the subjects [50,51]. Mixed forests form a stand structure with multiple layers and a thick canopy and have more abundant understory vegetation, thus, the beauty of the mixed forest is higher than that of the pure forests [52]. Comparing the city environment with the forest environments, “fascination” showed significant changes in both forest sites and city sites. This finding is consistent with ART, which posits that the attention-grabbing qualities of a natural environment are the core element of its restorative potential [6,19]. ART may offer some additional interpretation, as it states that a more natural environment tends to afford a restoration experience in the sense of being away, fascinated, coherent, and compatible.

### 4.2. Effects of Different Environments on Physiological Health

The results of the study showed significantly different levels of SBP, DBP, and HR restoration between the urban site and the forest sites, which is consistent with the results of previous studies that natural environments exert greater physiological restorative impacts than do built environments [53,54,55]. Forests are superior because they provide a special interior forest climate with reduced air temperature, high air purity and humidity, and special light conditions. These climatic factors are beneficial to health and relieve the respiratory tract and the thermoregulation system [56]. In addition, in line with our expectations, we found significant difference in the mean level of SBP, DBP, and HR restoration of subjects among three different types of forests, with strongest recovery in the mixed forest. This may be because a natural environment with rich sensory stimulation can easily arouse the biophilic emotion of people who love nature and desire to be subordinate to nature. The positive reaction of the body is more obvious in scenes with rich sensory stimulation [57]. In particular, the restorative qualities may be more perceptible in the mixed forest, which has a more complex tree composition.

### 4.3. Effects of Different Environments on Psychological Well-Being

From the results, it was found that forest environments had a better restorative effect on ROS, SVS, and WEMWBS than did the urban environment. This is consistent with the results of previous studies, which confirmed that values in the ROS and SVS increased because of forest exposure [31,48]. One of the most important factors is the peace and quiet in forests, which is essential for mental recovery in a time of acoustic stimulus overload in urban settings. Moreover, there were significant difference in ROS, SVS, and WEMWBS in three forests. The coniferous forest had the strongest effects on increase of ROS and WEMWBS, which may be related to the volatile organic compounds (VOCs) emitted by trees and plants. It has been confirmed that the coniferous forest was more productive in the emission of phytoncide [58], which is beneficial for reducing mental fatigue, improving cognitive performance, and inducing relaxation [59]. Environmental characteristics such as moderate complexity and depth are among the most important mechanisms following the SRT, which also explains why the coniferous forest in this study could provide a greater perceived psychological restoration experience than the others [60].

### 4.4. The Relationships between Restorative Perception of Environment and Physiological and Psychological Restoration

The reductions in SBP, DBP, and HR as well as the increments in ROS, SVS, and WEMWBS measured in the three forests may reflect the restorative capacity of forest environments. Additionally, the results of the physiological and psychological changes were significantly positive correlated with the PRS and the four dimensions of the PRS. These findings indicate that a higher perceived restoration assessment in the green environments and the significant correlations with the perceived level of restoration in these areas are consistent [61]. To a certain extent, this conclusion enriches the evidence of the meaning of perception of nature’s effect on health. Combining environmental characteristic assessment with changes in the physiological and psychological response will help us to fully understand the restorative effect of greenness and the mechanisms that benefit from it. In addition, it is worth noting that the result of our study showing correlation is significant, but the correlation coefficient is weak. To some degree, it means that the correlations are not strong overall.

### 4.5. Limitations

The study investigated the relationship between the perceived restorative power of the environment and the physiological and psychological restoration effects in young people of different types of forests. The results of our study confirm previous findings that forest environments have more positive impacts on the physiological and psychological characteristics of the subjects than urban environments and provide a novel exploration of the forest’s restorative effects. Nevertheless, there are some limitations in this study. First, we only explored the restoration effects of a short-term forest exposure on the participant’s health but did not examine the sustainability of the restorative effects. It is necessary to examine the duration of the forest exposure’s impacts by specific follow-up assessments. Second, only university students were involved in this study. Future studies should explore how forest exposure influences subjects of different socio-demographic backgrounds. Third, this study was carried out in summer, and the change of season is an important factor for forest landscapes and environments. Thus, it is important to examine how different seasons will affect the physiological and psychological parameters of the subjects. Fourth, the physiological measurements that were used had some limitations, for example, heart rate can be influenced by many different factors (including walking). Fifth, the extensive nature of the task might have influenced the results to some degree as all participants completed three questionnaires three times. In general, future research needs to consider the restorative effects of different natural environments with different characteristics of landscape and environments on subjects with special needs or preferences.

## 5. Conclusions

We examined the relationship between the restorative perception of the environment assessed via the Perceived Restoration Scale (PRS) and the physiological and psychological restoration achieved by experience of the forest environment. Results showed significant differences in the assessment of the restorative perception of the three types of forests, with the highest level in the mixed forest, followed by the coniferous forest and the deciduous forest. In addition, all types of forests had a stronger restorative effect than the urban environment did, as assessed by reductions in SBP, DBP, and HR and increments in ROS, SVS, and WEMWBS. We found significant decreases in blood pressure and heart rate and increases in SVS in the mixed forest, while ROS and WEMWBS significantly improved in the coniferous forest, which suggests that forest types and tree species composition are closely associated with forest therapy potential. In addition, physiological and psychological changes were significantly positively correlated with the PRS and the four dimensions of the PRS. These findings indicate that a higher perceived restoration experience in the green environments and the significant correlations with the perceived level of restoration in these areas are consistent. Exploring the differences in the restoration effects of different forest types can effectively support the design and management of future healing forest environments. These findings provide practical evidence for forest therapy that can maximize the restorative effects of forests.

## Figures and Tables

**Table 1:** Forest characteristics in forest sites.

Forest Site	Mixed Forest	Deciduous Forest	Coniferous Forest
Dominant tree species	*Pinus tabuliformis* *Quercus mongolica*	*Cotinus coggygria*	*Platycladus orientalis*
Tree height (m)	7 ± 1.22	5 ± 1.00	8 ± 0.81
Diameter breast height (cm)	22 ± 3.67	15 ± 3.35	16 ± 1.81
Canopy closure (%)	0.7 ± 0.03	0.55 ± 0.01	0.6 ± 0.05
Visual penetration through stand (m)	40 ± 1.47	25 ± 3.74	45 ± 1.23
Stand density (trees ha^−1^)	525 ± 11	664 ± 10	825 ± 8

Data are presented as the means ± SD.

**Table 2:** Total and dimensional scores of PRS.

	Scores of PRS	Dimensional Scores of PRS			
		Being Away	Fascination	Extent	Compatibility
CF	4.29 ± 1.06	5.01 ± 0.96	3.83 ± 1.02	4.81 ± 0.81	3.89 ± 0.81
DF	4.25 ± 1.12	3.99 ± 1.17	4.17 ± 1.13	4.36 ± 1.11	4.53 ± 0.97
MF	4.84 ± 0.95	4.74 ± 1.05	4.85 ± 0.96	4.73 ± 0.91	5.03 ± 0.82
CK	1.56 ± 1.13	1.23 ± 1.00	1.91 ± 1.29	1.24 ± 0.78	1.57 ± 1.05

Data are presented as the means ± SD. CF = coniferous forest, DF = deciduous forest, MF = mixed forest, CK = city site.

**Table 3:** Mean and standard deviation (SD) of physiological parameters (systolic blood pressure (SBP), diastolic blood pressure (DBP), and heart rate (HR)) in the four experimental sites.

		City Site		Mixed Forest		Deciduous Forest		Coniferous Forest	
		Mean	SD	Mean	SD	Mean	SD	Mean	SD
SBP	Baseline (TO)	113.30	4.71	117.10	4.44	114.33	6.48	116.07	5.35
		116.13	7.21	107.10	7.88	108.72	7.30	113.73	7.19
DBP	Baseline (TO)	66.70	4.24	70.77	5.12	68.63	5.50	71.30	4.69
		68.50	5.90	64.70	6.74	65.27	5.731	66.60	5.83
HR	Baseline (TO)	79.70	4.09	82.07	3.61	78.60	3.38	80.63	3.01
		81.17	4.60	76.92	5.66	75.90	4.65	78.53	5.67

**Table 4:** Mean and standard deviation (SD) of psychological parameters (ROS, SVS, and WEMWBS) in the four experimental sites.

		City Site		Mixed Forest		Deciduous Forest		Coniferous Forest	
		Mean	SD	Mean	SD	Mean	SD	Mean	SD
ROS	Baseline (TO)	2.03	0.75	2.21	0.78	2.15	0.76	1.79	0.81
		1.51	0.97	4.24	1.31	4.14	1.21	4.01	1.27
SVS	Baseline (TO)	1.83	1.03	1.74	0.88	1.80	0.95	1.97	0.91
		0.95	0.72	4.60	1.07	4.35	1.22	3.94	1.42
WEMWBS	Baseline (TO)	1.86	0.710	1.93	0.715	2.01	0.72	1.89	0.73
		1.57	1.04	2.95	0.89	2.95	0.92	3.02	0.87

**Table 5:** Spearman rank correlations between the PRS and four sub-scales of PRS and the SBP, DBP, and HR.

		PRS	Four Sub-Scales of PRS			
			Being Away	Fascination	Extent	Compatibility
SBP	Spearman Correlation	−0.506 **	−0.370 **	−0.468 **	−0.327 **	−0.506 **
	Sig. (two-tailed)	0.000	0.000	0.000	0.000	0.000
DBP	Spearman Correlation	−0.352 **	−0.328 **	−0.345 **	−0.373 **	−0.352 **
	Sig. (two-tailed)	0.000	0.000	0.000	0.000	0.000
HR	Spearman Correlation	−0.347 **	−0.258 **	−0.328 **	−0.357 **	−0.347 **
	Sig. (two-tailed)	0.000	0.000	0.000	0.000	0.000

** Correlation is significant at the *p* < 0.01 level (two-tailed).

**Table 6:** Spearman rank correlations between the PRS and four sub-scales of PRS and the ROS, SVS, and WEMWBS.

		PRS	Four Sub-Scales of PRS			
			Being Away	Fascination	Extent	Compatibility
ROS	Spearman Correlation	0.541 **	0.737 **	0.570 **	0.538 **	0.541 **
	Sig. (two-tailed)	0.000	0.000	0.000	0.000	0.000
SVS	Spearman Correlation	0.708 **	0.678 **	0.673 **	0.542 **	0.708 **
	Sig. (two-tailed)	0.000	0.000	0.000	0.000	0.000
WEMWBS	Spearman Correlation	0.458 **	0.669 **	0.535 **	0.502 **	0.458 **
	Sig. (two-tailed)	0.000	0.000	0.000	0.000	0.000

** Correlation is significant at the *p* < 0.01 level (two-tailed).

## Data Availability

No publicly archived datasets, analyzed or generated, were used in this study.
